# Knowledge, Attitudes, and Practices of General Nurses, Specialist Nurses, Resident Nurses, and Nursing Students Regarding Informed Consent: A Scoping Review Protocol

**DOI:** 10.3390/nursrep16080272

**Published:** 2026-08-04

**Authors:** Airam Cabrera-Rodríguez, Jacqueline Schmidt-RioValle

**Affiliations:** 1 Doctoral Program in Clinical Medicine and Public Health, University of Granada, 18016 Granada, Spain; jschmidt@ugr.es; 2 Instituto de Investigación Biosanitaria (ibs.GRANADA), 18016 Granada, Spain; 3 Department of Nursing, Faculty of Medicine, Health and Sports, European University of Madrid, 28670 Madrid, Spain; 4 Department of Nursing, Faculty of Health Sciences, University of Granada, 18071 Granada, Spain

**Keywords:** bioethics, health knowledge, attitudes, practice, health law, informed consent, nursing ethics, nurse specialists, nursing students, nurse’s role, nursing

## Abstract

**Background**: Informed consent is an essential ethical and legal process that safeguards patient autonomy, integrity, and rights in healthcare. Although it has traditionally been associated with medical practice, the expanding scope of nursing practice and increased professional autonomy require a clearer understanding of nurses’ roles in this process. Given nurses’ continuous and close contact with patients, examining their knowledge, attitudes, and practices regarding informed consent is essential to ensure ethical standards and patient safety. **Objectives**: This scoping review will map and synthesize the available evidence on the knowledge, attitudes, and practices of general nurses, specialist nurses, resident nurses, and nursing students in relation to informed consent. **Methods**: This review will follow the Joanna Briggs Institute methodology for scoping reviews and will be guided by the PRISMA-ScR extension. A comprehensive search strategy will be applied to MEDLINE, CINAHL, Web of Science, Scopus, and Cuidatge databases. Quantitative, qualitative, and mixed-methods studies addressing nurses’ knowledge, attitudes, and practices regarding informed consent will be included. Discussion papers, reflective articles, commentaries, letters to the editor, opinion articles, books, book chapters, gray literature, and literature reviews will be excluded. Study selection and data extraction will be performed systematically, and findings will be synthesized descriptively and presented through tables and narrative analysis. **Conclusions**: This protocol outlines a transparent and reproducible methodology for a scoping review. It will map evidence on nurses’ and nursing students’ knowledge, attitudes and practices regarding informed consent; it will summarize the existing evidence as well. This study was prospectively registered with the Open Science Framework (OSF) on 13 May 2026.

## 1. Introduction

Informed consent (IC) is a formal and progressive procedure of ethical and legal requirements [1]. Through this process, an individual freely, voluntarily, and knowingly authorizes a clinical intervention, based on the prior provision of clear, understandable, and individualized information [1,2,3].

Currently, IC represents a fundamental element of the therapeutic relationship, supporting the patient’s right to make decisions about their own body and ensuring respect for their physical and moral integrity [1,2]. Traditionally, IC has been closely associated with medical practice [1]. However, the development, recognition, and increasing autonomy of other healthcare professions, such as nursing, physiotherapy, occupational therapy, and podiatry, make it necessary to analyze how these professionals engage with IC [2].

Particularly, nurses are healthcare professionals who maintain the closest and most continuous contact with patients, providing direct care across all healthcare settings [4,5,6]. They deliver comprehensive, autonomous, and collaborative care to individuals, families, and communities throughout patients’ lifespan and in different contexts [5,6,7,8]. Their role includes health promotion, disease prevention, treatment, and rehabilitation, as well as continuous support during health and illness processes [9]. In addition, nurses carry out clinical, educational, management, and research activities based on scientific knowledge, evidence-based practice, and professional ethics [6,9,10]. All these responsibilities require nurses to perform according to internationally recognized professional values and standards [4,5,9,10].

In this context, the International Council of Nurses’ Code of Ethics establishes that nurses have the responsibility to ensure the right to information and to obtain IC, which is a legitimizing element of clinical practice (Art. 1.3 ICN) [9]. They must also protect the patient’s autonomy in procedures carried out by other healthcare professionals (Art. 2.7 ICN) [9]. This highlights the importance of analyzing nurses’ knowledge, attitudes, and practices in relation to IC.

Despite the differences between the countries’ legal requirements, IC is widely recognized as an essential component of ethical clinical practice. Written IC is typically required for certain interventions, particularly invasive or high-risk procedures, depending on national regulations [2,4,5,6,9,10]. However, several studies have shown that nurses do not always acknowledge the importance of this requirement [2,4]. For example, Lamont et al. (2017) found that only two-thirds of nurses considered that all interventions or treatments require IC [11]. Similarly, Fernández-Garrido et al. (2009) reported that only 60% of nurses correctly identified the format in which patients should provide IC [12]. Specifically, the qualitative study by Aveyard et al. (2022) showed that, before nursing care, patients often granted implicit and informal consent rather than a formal and explicit one [13].

On the other hand, the study by Nortes et al. (2001) showed that, although 100% of nurses considered it essential to dedicate time and space to IC, 40% were unaware of whether it was a legally required document [14]. Along these lines, Akyüz et al. (2018) demonstrated that while 90.2% of nurses informed patients before performing nursing interventions, only 32.6% obtained IC [15]. Furthermore, in the same study by Nortes et al. (2001), 81.3% believed that a paternalistic dynamic in the professional–patient relationship represents a barrier to IC [14]. Similarly, Arenas López et al. (2012) found that nurses were more likely than physicians to believe that a patient’s wish not to be informed should be respected (96.2% vs. 89.9%) [16]. This may be explained by the traditionally paternalistic nature of the physician–patient relationship and by the role of nurses as patient advocates and interpreters [17,18,19].

Among nursing students, the findings reported by Bautista-Espinel (2015) are concerning: 53% believed that, from a nursing perspective, whether a patient accepts or refuses a care intervention or treatment should not be a relevant factor in professional practice [20]. Similarly, in the study by Galván-Meléndez et al. (2016), only 55.6% of resident nurses reported having been granted IC at any given time from a patient prior to a clinical intervention [21].

In the current context of healthcare, the effective implementation of IC continues to present several challenges, including limitations in the information provided, insufficient patient understanding, and difficulties ensuring voluntary decision-making. Nurses, as key professionals in direct care and patient support, play an important role in verifying and ensuring the quality of this process, contributing to patient safety and the legal validity of clinical interventions.

A preliminary search was conducted in PROSPERO, PubMed, the Cochrane Database of Systematic Reviews, and JBI Evidence Synthesis, and no current or ongoing scoping reviews or systematic reviews on this topic were identified. Between 21 January 2026 and 12 February 2026, the research members carried out initial independent research. Later, it was performed jointly, using combinations of the main concepts of the review (informed consent, nurses, nursing students, knowledge, attitudes, and practices). No language or publication date filters were applied.

Despite the ethical and legal relevance of IC, the available evidence on nurses’ knowledge, attitudes, and practices remains fragmented; it has primarily focused on specific professional groups, healthcare settings, or isolated aspects of the IC process. To date, only the CONOSER pilot study has simultaneously examined the knowledge, attitudes, and practices of IC among general nurses, specialist nurses, and resident nurses [2]. The results are similar to those of other studies previously discussed [11,12,13,14,15,16,17,18,19,20,21]. However, this study was limited to primary care nurses in a single geographic area and did not include nursing students, leaving the broader body of evidence across different nursing profiles, healthcare contexts, and educational stages unexplored. Therefore, this scoping review protocol is aimed at systematically mapping the existing evidence. It also provides a comprehensive overview of the scientific literature in this field.

## 2. Materials and Methods

The proposed scoping review will be conducted in accordance with the JBI methodology for scoping reviews and reported in line with the Preferred Reporting Items for Systematic Reviews and Meta-Analyses extension for Scoping Reviews (PRISMA-ScR) [22,23].

### 2.1. Review Question

What are the knowledge, attitudes, and practices among general nurses, specialist nurses, resident nurses, and nursing students regarding informed consent in the different professional contexts?

### 2.2. Inclusion Criteria

#### 2.2.1. Participants

This scoping review protocol will consider studies that examine general nurses’, specialist nurses’, resident nurses’, and nursing students’ knowledge, attitudes, and/or practices. They will be looked into in relation to IC, regardless of their level of clinical autonomy or legal responsibility within the IC process.

The studies will be considered regardless of their scope of professional activity (primary health care and hospital health care), the roles they carry out (clinical, management, training, and research), and their category or specialty (general nurses, specialist nurses, resident nurses, and nursing students). The international scope of review calls for the identification of different categories. In this case, they will be professional titles, educational status, and definitions reported by the primary studies. It acknowledges that nursing education, specialization pathways, and regulatory frameworks differ across countries. For the purposes of this review, the following roles are considered:

- General nurses: registered nurses without a formally recognized nursing specialty.
- Specialist nurses: registered nurses holding a recognized specialty qualification or equivalent advanced specialty training.
- Resident nurses: graduate nurses undertaking a structured specialist residency or equivalent postgraduate clinical training program.
- Nursing students: individuals enrolled in an official undergraduate nursing education program.

The inclusion of the aforementioned nursing categories is intentional and consistent with the objective of this scoping review protocol. It is to comprehensively map the available evidence across the continuum of nursing education and professional practice rather than to compare homogeneous populations. Consequently, findings will be synthesized and presented separately according to each nursing category; this will facilitate interpretation while preserving the breadth of the evidence.

Studies including mixed nursing categories (general nurses, specialist nurses, resident nurses, and nursing students) will be included even when the findings cannot be disaggregated by nursing subgroup. In these cases, the results will be presented as mixed nursing categories. However, studies including nurses together with other healthcare professionals will only be eligible if data relating to the nursing population can be extracted separately.

#### 2.2.2. Concept

This review will consider studies that explore the knowledge, attitudes, and practices of nurses in relation to the IC in the health care sector. For the purposes of this scoping review protocol, a widely used healthcare conceptual framework of the knowledge, attitudes, and practices (KAP) [24,25] has been defined as follows:

- Knowledge: Theoretical understanding of informed consent, including ethical principles, legal requirements, and procedural elements.
- Attitudes: Beliefs, perceptions, and ethical stances regarding informed consent.
- Practices: Behaviors related to the implementation of informed consent, such as providing information, assessing patient understanding, documenting the process, and supporting patient decision-making.

For the purposes of this scoping review protocol, IC will be considered as a broad concept encompassing all forms of consent described in the literature; explicit and implied consent, as well as verbal and written ones, will be included according to the definitions adopted in each primary study.

Furthermore, IC will encompass situations in which general nurses, specialist nurses, resident nurses, or nursing students are involved. The contexts of those situations are clinical care, education, research, or management, including providing information, facilitating patient understanding, supporting decision-making, and obtaining or documenting consent. They apply depending on their professional role and the context described in each primary study.

Studies assessing one, two, or all three domains (knowledge, attitudes, and/or practices) related to IC will be eligible for inclusion. During data extraction, each domain will be recorded independently according to the outcomes reported in the primary studies.

#### 2.2.3. Context

The scoping review will consider studies conducted in any healthcare, educational, management, or research setting in which general nurses, specialist nurses, resident nurses, or nursing students participate in, or are expected to participate in, IC processes. The review will not be restricted by healthcare level, clinical specialty, geographical region, or legal framework. However, studies will only be eligible if they specifically investigate general nurses’, specialist nurses’, resident nurses’ or nursing students’ knowledge, attitudes, and/or practices regarding IC. Studies focusing exclusively on IC from the perspective of patients or other healthcare professionals, or addressing research ethics or patient rights without examining nurses’ or nursing students’ involvement in IC, will be excluded.

This scoping review protocol intentionally includes the different professional contexts in which IC may arise, including clinical care, education, research, and management. This broad contextual scope is consistent with JBI guidance on scoping reviews, whose purpose is to comprehensively map the available evidence on complex topics. To preserve conceptual clarity, the findings will be synthesized and presented separately according to the professional context in which IC is addressed.

#### 2.2.4. Types of Sources

This scoping review will consider designs of quantitative, qualitative and mixed-method studies. The quantitative methods will include descriptive, analytic-observational, quasi-experimental and experimental designs. The qualitative designs may include phenomenological, narrative, action research, documentary, grounded theory, analytic induction and feminist research designs. Considered mixed-method studies include concurrent, sequential, conversion and integration designs.

Papers excluded are those of discussion, reflection, comments, letters to the editor, opinion articles, books and book chapters, conference proceedings, presentations, lectures and oral or written communications. This exclusion of gray literature is motivated by the need to ensure scientific rigor, prioritize methodological quality, limit the research team’s working time, and manage resources realistically. Furthermore, the primary aim of this scoping review is to synthesize evidence derived from peer-reviewed empirical studies that have undergone formal scientific evaluation. Restricting the review to peer-reviewed publications is intended to enhance the methodological consistency and comparability of the included evidence, thereby facilitating a more robust synthesis of nurses’ knowledge, attitudes, and practices regarding IC. This is particularly relevant because studies exploring knowledge, attitudes, and practices frequently use observational and survey-based designs, for which peer-reviewed publications generally provide more comprehensive methodological reporting, thereby supporting consistent data extraction and comparison across studies. This decision also reflects the need to conduct the review within the available resources.

Likewise, in order to avoid data duplication, literature reviews (meta-analysis, systematic reviews, narrative reviews, integrative reviews, umbrella reviews, scoping reviews, and meta-synthesis) will be excluded. However, their reference lists will be screened (backward citation tracking) to identify potentially relevant primary studies. In addition, forward citation tracking of the studies included in the review will be conducted to identify further eligible studies.

### 2.3. Search Strategy

The search strategy will be developed following a 3-step approach, with the aim of identifying the most relevant published evidence about the knowledge, attitudes, and practices of nurses in relation to IC.

In the first stage, a preliminary search was carried out in MEDLINE (PubMed) to find relevant articles, as well as to analyze the keywords used in titles, summaries, abstracts, and MeSH terms of the most relevant studies. This initial search was carried out in close collaboration with an academic librarian specialized in health sciences. The keywords and the topic headings identified were combined with synonyms and related terms, developing a complex array of search terms. The complete search strategy for MEDLINE will be provided in Table 1, including all the MeSH terms and keywords used.

The preliminary MEDLINE search strategy was pilot-tested to ensure its ability to retrieve key studies relevant to the review question. The revised strategy successfully identified several sentinel articles previously known to the review team, including Aveyard et al. (2022) [13], Akyüz et al. (2019) [15], Axson et al. (2019) [18], and Cabrera-Rodríguez et al. (2023) [2]. These results support the adequacy and sensitivity of the search strategy.

In the second stage, the search strategy applied to MEDLINE was adapted to the rest of the databases, considering their particularities and indexation systems. The refined strategy for the main academic databases in health science, including MEDLINE (via PubMed), CINAHL (via EBSCOhost), Web of Science (via Clarivate Analytics), Scopus (via Elsevier), and Cuidatge (via Universitat Rovira i Virgili), can be consulted in Appendix A.

In the final stage, the full search strategy will be applied to the aforementioned databases. Backward citation tracking will be performed by screening the reference lists of the included studies and relevant reviews to identify additional potentially eligible studies. In addition, forward citation tracking of the included studies will be undertaken to identify further relevant evidence.

Studies published from 2000 onwards will be considered for inclusion. This time frame was selected following a period of major international developments in the ethical and legal framework governing IC. The adoption of the Convention on Human Rights and Biomedicine (Oviedo Convention, 1997) was the first legally binding international instrument establishing that any health-related intervention requires the free and IC of the individual [26]. During the early 21st century, these principles were progressively incorporated into national legislation and professional practice in many countries, including Spain through Law 41/2002, of 14 November, Basic Regulator of the Patient’s Autonomy and Rights and Obligations Regarding Clinical Information and Documentation (Ley 41/2002, de 14 de noviembre, básica reguladora de la autonomía del paciente y de derechos y obligaciones en materia de información y documentación clínica) [3]. Restricting the review to studies published since 2000 also ensures that the evidence reflects contemporary models of IC and current nursing practice, while providing a sufficiently broad time frame to comprehensively map the literature published throughout the 21st century. In addition, only studies published in English and Spanish will be considered. This decision was made to ensure accurate study selection, data extraction, methodological appraisal, and interpretation by reviewers with full proficiency in these languages, thereby avoiding potential inaccuracies associated with external translation. This is particularly relevant in a review of informed consent, where ethical and legal concepts may be interpreted differently across languages and jurisdictions, making conceptual accuracy essential throughout the review process. English was selected because it is the predominant language of international scientific publication, whereas Spanish was included to capture relevant evidence from Spanish-speaking settings.

### 2.4. Source of Evidence Selection

Following the search, all identified records will be collated and uploaded into Zotero, and duplicates will be removed. After ensuring there are no duplicates, the library will then be imported into Covidence (Veritas Health Innovation, Melbourne, Australia) to manage the screening process [27]. In this context, Zotero (version 9.0.5, USA, 2006) will be used as a reference management software and for the initial duplicate removal. Conversely, Covidence will be used to manage title and abstract selection, full-text review, and study selection.

Before formal selection, a pilot test will be conducted in which two reviewers will independently test the eligibility criteria by examining a random sample of records. The sample consists of 25 scientific articles. Any discrepancies will be discussed and resolved by consensus, and if necessary, the eligibility criteria will be refined to ensure a consistent interpretation. The formal selection process will begin once the discrepancies have been discussed and a common interpretation of the eligibility criteria has been reached.

Finally, two reviewers will independently examine the titles and abstracts, followed by an independent assessment of the full text of potentially eligible studies. Discrepancies at any stage will be resolved through discussion or, if necessary, by consulting a third reviewer. Reasons for exclusion at the full-text stage will be recorded using three predefined categories (population, concept, and context) and reported in the final review. The results will be reported in the final review, accompanied by a PRISMA flow diagram [28].

### 2.5. Data Extraction

Data will be extracted independently by two reviewers using a standardized data extraction instrument developed by the review team (Table 2). The extracted information will include study identification (author, year, journal, and language), study characteristics (country, aims, design, methodological approach, healthcare setting, and clinical field), participant characteristics (nursing population, sample size, and professional role), and characteristics of IC. It will also include its definition or conceptualization (when reported), the type of IC addressed, the clinical or professional context, and the legal or regulatory framework, where available.

In addition, data related to the review question will be extracted, including whether the study assessed nurses’ or nursing students’ knowledge, attitudes, and/or practices regarding IC, the instruments or methods used for their assessment, the principal findings, and any reported barriers, facilitators, or implications for nursing education and clinical practice. The data extraction instrument has been designed to enable the independent extraction of information related to each of the three domains (knowledge, attitudes, and practices). Therefore, studies addressing one, two, or all three domains will be eligible, and each reported outcome will be extracted separately.

The draft data extraction instrument may be refined during the review process to ensure that all relevant information required to address the review objectives is captured. Any modifications to the extraction instrument will be agreed upon by consensus between the reviewers before implementation, and all changes will be documented, justified, and reported in the final scoping review. If substantial modifications are introduced after data extraction has commenced, data from studies extracted previously will be reviewed and updated; the revised version of the instrument will be used to ensure consistency across all included studies. Disagreements between reviewers will be resolved through discussion or, when necessary, by consultation with a third reviewer. Where required, corresponding authors will be contacted up to twice to request missing or additional information.

### 2.6. Methodological Quality Appraisal and Presentation of Results

The methodological quality of all included studies will be independently assessed by two reviewers. They will use the appropriate Critical Appraisal Tools of JBI, according to the design of each study [29]. Any disagreements will be resolved through discussion or, when necessary, by consultation with a third reviewer. The purpose of this appraisal is to provide readers with additional information regarding the methodological characteristics of the available evidence. In addition, the interpretation of the review findings, like the risk of bias of the studies included, will be facilitated. The results of the methodological appraisal will not be used as criteria for excluding studies or to determine the weight assigned to individual studies within the review.

The results of the scoping review will be reported in accordance with the PRISMA Extension for Scoping Reviews (PRISMA-ScR) guidelines. They will be presented using tables, figures, and a narrative synthesis aligned with the review objectives [28]. Where appropriate, descriptive frequencies will be used to summarize the distribution of study characteristics. The evidence will be organized according to the knowledge, attitudes, and practices addressed by each study. Whenever possible, the evidence will be further categorized by nursing categories (general nurses, specialist nurses, resident nurses, and nursing students), professional context (clinical care, education, research, and management), healthcare setting, legal or regulatory framework, and study design. Reported barriers, facilitators, and implications for nursing education and clinical practice will be synthesized narratively; the aim is to identify patterns and gaps in the available evidence. The results of the methodological quality appraisal will be presented alongside the evidence map to facilitate interpretation; however, it will not be used to exclude studies or determine the weight assigned to individual findings.

### 2.7. Protocol Registration

This protocol has been registered in the Open Science Framework (OSF). Any amendments made after protocol publication will be documented in the OSF registration (OSF registration: https://osf.io/72b9r), including the date of the modification and the rationale for the change. These amendments will also be transparently reported and justified in the final scoping review publication. Any protocol deviations will likewise be reported and justified.

## 3. Discussion

This protocol describes a transparent and reproducible methodological framework for conducting a scoping review that will collate and map the available scientific evidence on the knowledge, attitudes, and practices of general nurses, specialist nurses, resident nurses, and nursing students regarding IC. The broad and systematic approach seeks to provide a comprehensive overview of the existing literature across different nursing categories, healthcare settings, and professional contexts.

Thus, this protocol lays the groundwork for the future scoping review, which has the potential to identify differences in knowledge, attitudes, and practices regarding IC among nursing populations or care settings. The future review may also contribute to identifying inconsistencies between theoretical knowledge and clinical implementation, where such evidence exists. Consequently, areas where educational and organizational interventions are needed could be identified.

Several limitations should be considered when interpreting the findings of this scoping review protocol. First, limiting the review to peer-reviewed empirical studies and excluding gray literature may prevent the inclusion of relevant evidence, such as institutional reports, professional guidance, or emerging research not yet published in scientific journals. Likewise, although the search strategy has been designed to maximize the identification of relevant studies, restricting the search to the selected bibliographic databases may result in the omission of potentially eligible publications. In addition, only studies published in English and Spanish will be considered. While this decision reflects the language proficiency of the review team and facilitates accurate screening and data extraction, it may exclude relevant evidence published in other languages. Consequently, studies published in languages other than English or Spanish, particularly those conducted in countries with different legal frameworks, healthcare systems, and sociocultural approaches to IC may be underrepresented. This may limit the global representativeness of the review. Finally, variations in the conceptualization of IC, together with differences in study designs and healthcare contexts, may limit the comparability of the included studies. Nevertheless, this scoping review protocol is expected to allow mapping of available evidence and provide a comprehensive overview of current literature.

## 4. Conclusions

This protocol describes a transparent and reproducible methodology for a scoping review that will map the available scientific evidence on the knowledge, attitudes, and practices of general nurses, specialist nurses, resident nurses, and nursing students regarding IC. Through the systematic collection of scientific literature under this protocol, the scoping review will provide an overview of the scope, characteristics, and distribution of the available evidence, including the populations studied, care settings, and methodological approaches employed. It is hoped that the findings of the scoping review will provide a useful evidence base to support future research, nursing education, and the development of strategies and initiatives aimed at strengthening IC practices.

## Figures and Tables

**Table 1:** Search strategy in MEDLINE.

MEDLINE (Via PubMed). Search Conducted: 14 July 2026		
Search	Query	Records Retrieved
1	(“Informed Consent” [Mesh] OR “informed consent” [tiab] OR “consent process*” [tiab] OR “informed consent process*” [tiab] OR “consent discussion*” [tiab] OR “informed decision*” [tiab] OR “valid consent” [tiab] OR “patient consent” [tiab]) AND (“Nurses” [Mesh] OR “Nursing” [Mesh] OR “Students, Nursing” [Mesh] OR nurs* [tiab] OR “registered nurse*” [tiab] OR “general nurse*” [tiab] OR “specialist nurse*” [tiab] OR “clinical nurse specialist*” [tiab] OR “resident nurse*” [tiab] OR “nursing resident*” [tiab] OR “nursing student*” [tiab] OR “student nurse*” [tiab]) AND (“Knowledge” [Mesh] OR knowledge [tiab] OR awareness [tiab] OR understanding [tiab] OR familiarity [tiab] OR “Attitude of Health Personnel” [Mesh] OR attitude* [tiab] OR perception* [tiab] OR opinion* [tiab] OR belief* [tiab] OR viewpoint* [tiab] OR “Practice” [Mesh] OR practice* [tiab] OR implementation [tiab] OR behaviour [tiab] OR behavior [tiab] OR compliance [tiab] OR “Decision Making” [Mesh] OR “decision making” [tiab] OR “Personal Autonomy” [Mesh] OR autonomy [tiab])	2833

* Publication date limited from 2000 to 2026, in Spanish and English language and human studies.

**Table 2:** Draft data extraction instrument.

Data Extraction Item	Response Field
**Study identification**	
First author:	
Year of publication	
Journal:	
Language:	☐ English ☐ Spanish
**Study characteristics**	
Country:	
Aim(s) of the study:	
Study design:	
Methodological approach:	☐ Quantitative ☐ Qualitative ☐ Mixed-methods
Healthcare setting:	
Clinical specialty/field:	
**Participant characteristics**	
Nursing population:	☐ General ☐ Specialist ☐ Resident ☐ Students ☐ Mixed nursing categories
Professional role addressed:	☐ Clinical ☐ Management ☐ Education/training ☐ Research
Sample size:	
**Characteristics of IC**	
Definition of IC:	
Type of IC:	☐ Verbal ☐ Written ☐ Implied ☐ Explicit ☐ Not specified
Health procedure:	
Legal or regulatory framework:	
**Knowledge, attitudes and practices:**	
Analyses of IC knowledge:	☐ Yes ☐ No
Analyses of IC attitudes:	☐ Yes ☐ No
Analyses of IC practices:	☐ Yes ☐ No
Measurement instrument/method:	
Main outcomes/findings:	
Barriers identified:	
Facilitators identified:	
Educational or practice implications:	
**JBI Critical Appraisal Tool**	
Methodological quality appraisal:	
**Additional information**	
Further comments and observations by the reviewers:	

## Data Availability

As a scoping review protocol, no new data were created or analyzed in this study.

## Public Involvement Statement

No public involvement in any aspect of this research.

## Guidelines and Standards Statement

This manuscript was drafted against the Preferred Reporting Items for Systematic Reviews and Meta-Analyses extension for Scoping Reviews (PRISMA-ScR) guidelines.

## Abbreviations

The following abbreviations are used in this manuscript:

IC	Informed Consent
ICN	International Council of Nurses
JBI	Joanna Briggs Institute
PRISMA-ScR	Preferred Reporting Items for Systematic Reviews and Meta-Analyses extension for Scoping Reviews
OSF	Open Science Framework
MeSH	Medical Subject Headings

## Appendix A

**Table A1:** Search strategy developed for databases in the scoping review.

Databases	Search Strategy (Search Conducted: 21 July 2026)	Records Retrieved
MEDLINE (Via PubMed)	(“Informed Consent” [Mesh] OR “informed consent” [tiab] OR “consent process*” [tiab] OR “informed consent process*” [tiab] OR “consent discussion*” [tiab] OR “informed decision*” [tiab] OR “valid consent” [tiab] OR “patient consent” [tiab]) AND (“Nurses” [Mesh] OR “Nursing” [Mesh] OR “Students, Nursing” [Mesh] OR nurs* [tiab] OR “registered nurse*” [tiab] OR “general nurse*” [tiab] OR “specialist nurse*” [tiab] OR “clinical nurse specialist*” [tiab] OR “resident nurse*” [tiab] OR “nursing resident*” [tiab] OR “nursing student*” [tiab] OR “student nurse*” [tiab]) AND (“Knowledge” [Mesh] OR knowledge [tiab] OR awareness [tiab] OR understanding [tiab] OR familiarity [tiab] OR “Attitude of Health Personnel” [Mesh] OR attitude* [tiab] OR perception* [tiab] OR opinion* [tiab] OR belief* [tiab] OR viewpoint* [tiab] OR “Practice” [Mesh] OR practice* [tiab] OR implementation [tiab] OR behaviour [tiab] OR behavior [tiab] OR compliance [tiab] OR “Decision Making” [Mesh] OR “decision making” [tiab] OR “Personal Autonomy” [Mesh] OR autonomy [tiab])	2838
Web of Science (WOS) (Via Clarivate)	((“informed consent” OR “consent process*” OR “informed consent process*” OR “consent discussion*” OR “patient consent” OR “valid consent”) AND (nurs* OR “registered nurse*” OR “general nurse*” OR “specialist nurse*” OR “clinical nurse specialist*” OR “resident nurse*” OR “nursing resident*” OR “student nurse*” OR “nursing student*”) AND (knowledge OR awareness OR understanding OR familiarity OR attitude* OR perception* OR opinion* OR belief* OR viewpoint* OR practice* OR implementation OR behaviour OR behavior OR compliance OR autonomy OR “decision making”))	2144
CINAHL (Via EBSCOhost)	((MH “Informed Consent+”) OR TI (“informed consent” OR “consent process*” OR “informed consent process*” OR “consent discussion*” OR “patient consent”) OR AB (“informed consent” OR “consent process*” OR “informed consent process*” OR “consent discussion*” OR “patient consent”)) AND ((MH “Nurses+”) OR (MH “Nursing+”) OR (MH “Students, Nursing+”) OR TI (nurs* OR “registered nurse*” OR “specialist nurse*” OR “resident nurse*” OR “clinical nurse specialist*” OR “student nurse*”) OR AB (nurs* OR “registered nurse*” OR “specialist nurse*” OR “resident nurse*” OR “clinical nurse specialist*” OR “student nurse*”)) AND ((MH “Knowledge+”) OR (MH “Attitude of Health Personnel+”) OR (MH “Practice Patterns+”) OR TI (knowledge OR awareness OR understanding OR familiarity OR attitude* OR perception* OR opinion* OR belief* OR practice* OR implementation OR behaviour OR behavior OR compliance OR autonomy) OR AB (knowledge OR awareness OR understanding OR familiarity OR attitude* OR perception* OR opinion* OR belief* OR practice* OR implementation OR behaviour OR behavior OR compliance OR autonomy))	1214
Scopus (Via Elsevier)	((“informed consent” OR “consent process*” OR “informed consent process*” OR “consent discussion*” OR “patient consent” OR “valid consent”) AND (nurs* OR “registered nurse*” OR “general nurse*” OR “specialist nurse*” OR “clinical nurse specialist*” OR “resident nurse*” OR “nursing resident*” OR “student nurse*” OR “nursing student*”) AND (knowledge OR awareness OR understanding OR familiarity OR attitude* OR perception* OR opinion* OR belief* OR viewpoint* OR practice* OR implementation OR behaviour OR behavior OR compliance OR autonomy OR “decision making”))	5520
Cuidatge (Via Universitat Rovira i Virgili)	(“consentimiento informado” + consentimiento) * (enfermer $ + estudiant $)	25

* Publication date limited from 2000 to 2026, in Spanish and English language and human studies. $ Truncation for Cuidatge Database.
